# A two-way street: bridging implementation science and cultural adaptations of mental health treatments

**DOI:** 10.1186/1748-5908-8-90

**Published:** 2013-08-19

**Authors:** Leopoldo J Cabassa, Ana A Baumann

**Affiliations:** 1 School of Social Work, Columbia University, 1255 Amsterdam Ave, New York, NY, USA; 2 Department of Psychiatry, College of Physicians & Surgeons, Columbia University, 1051 Riverside Drive, New York, NY, USA; 3 George Warren Brown School of Social Work, Washington University in St. Louis, 600 S. Taylor, Suite 122, St. Louis, 63110, Missouri, MO, USA

**Keywords:** Cultural adaptation, Implementation science, Mental health care disparities

## Abstract

**Background:**

Racial and ethnic disparities in the United States exist along the entire continuum of mental health care, from access and use of services to the quality and outcomes of care. Efforts to address these inequities in mental health care have focused on adapting evidence-based treatments to clients’ diverse cultural backgrounds. Yet, like many evidence-based treatments, culturally adapted interventions remain largely unused in usual care settings. We propose that a viable avenue to address this critical question is to create a dialogue between the fields of implementation science and cultural adaptation. In this paper, we discuss how integrating these two fields can make significant contributions to reducing racial and ethnic disparities in mental health care.

**Discussion:**

The use of cultural adaptation models in implementation science can deepen the explicit attention to culture, particularly at the client and provider levels, in implementation studies making evidence-based treatments more responsive to the needs and preferences of diverse populations. The integration of both fields can help clarify and specify what to adapt in order to achieve optimal balance between adaptation and fidelity, and address important implementation outcomes (*e.g*., acceptability, appropriateness). A dialogue between both fields can help clarify the knowledge, skills and roles of who should facilitate the process of implementation, particularly when cultural adaptations are needed. The ecological perspective of implementation science provides an expanded lens to examine how contextual factors impact how treatments (adapted or not) are ultimately used and sustained in usual care settings. Integrating both fields can also help specify when in the implementation process adaptations may be considered in order to enhance the adoption and sustainability of evidence-based treatments.

**Summary:**

Implementation science and cultural adaptation bring valuable insights and methods to how and to what extent treatments and/or context should be customized to enhance the implementation of evidence-based treatments across settings and populations. Developing a two-way street between these two fields can provide a better avenue for moving the best available treatments into practice and for helping to reduce racial and ethnic disparities in mental health care.

## Introduction

Compared to non-Hispanic whites, racial and ethnic minorities in the U.S. are more likely to underutilize mental health services, discontinue treatments prematurely, and to receive care that is poor in quality even after adjusting for differences in educational levels, socioeconomic status, health insurance rates, and mental health needs [1,2]. Some of the efforts to address these disparities in mental health care have focused on adapting evidence-based treatments (EBT) to clients’ culture. As expressed more than a decade ago in the Surgeon’s General report on ‘Culture, Race, Ethnicity and Mental Health’ [2], ‘culture counts’ in mental health care, as it shapes how people seek help, engage in health behaviors, and how providers communicate with clients and deliver services. Culture is more than race and ethnicity, as it encompasses ‘a shared way of being and interacting’ [3] that shapes lifestyle patterns and structures human thoughts, emotions, interactions, social norms, and behaviors [4]. Culture is not a static set of values, norms and practices that reside solely within the individual; it is dynamic, as it is learned, transmitted and transformed by social interactions, conflicts, and power relations [5-8].

The basic assumption for adapting mental health treatments to client’s cultural backgrounds is that, by explicitly integrating cultural factors (*e.g*., language, cultural values, gender roles) into care, the relevance, acceptability, effectiveness and sustainability of treatments will be increased, and inequities in care will be narrowed [9]. Yet, like many EBTs in the U. S., these culturally adapted treatments remain largely unused in usual care settings. A central question to address this gap in mental care that remains unanswered is how to go about translating the best available knowledge of treatments in historically underserved communities to eliminate inequities in mental health care.

In this paper, we propose that a viable avenue to address this critical question is to bridge and create a dialogue between the fields of implementation science (IS) and cultural adaptations (CA). We begin by providing a rationale for integrating these two fields. We then discuss five critical areas that would benefit from this integration: making culture more visible in the implementation process; determining if adaptations are needed, examining what to adapt in order to balance fidelity and adaptation in the implementation of EBTs; examining who should drive the process of implementation and adaptation of EBTs in usual care settings; expanding the contextual lens to inform adaptations and implementation strategies; and examining when to adapt EBTs, if necessary, in the implementation process. For each of these areas, we present implications for research.

In this paper, we focus and draw mostly from studies conducted in the U.S. because of the persistent racial and ethnic mental health care disparities in this country and the expertise and experiences of the authors working in the U.S. context. Many of the points we discuss here could be used when implementing and adapting EBTs in other countries facing inequities in mental health care [10,11], but should be applied with caution given the unique cultural, social, historical and political forces that shape how EBTs were developed, and the new settings where these EBTs are being implemented. As IS and CA in mental health services research and practice are both growing and evolving fields, the ideas presented in this paper should be viewed as points of departure for future dialogues.

### Why integrate implementation science and cultural adaptations?

The implementation of mental health practice innovations into usual care settings is more than a technical endeavor of simply training or disseminating new knowledge to providers to deliver a new treatment [12]. It also entails social processes that are dynamic and highly dependent upon the context in which the innovation takes place [13]. As stipulated by Rogers, an ‘innovation [such as an EBT] almost never fits perfectly in the organization in which it is being embedded’ [14]. Implementation is a mutual adaptation process in which both the treatment that is being implemented and the organizations and stakeholders (*e.g*., consumers, providers, administrators) involved accommodate to the parameters of the new treatment and the exchange of knowledge, attitudes, social norms, and practices that occur throughout this process [13,15,16].

As presented in Table 1, both IS and CA fields contribute valuable insights into this transformation of treatments and context in moving research into practice, but tackle this process from different angles. IS views this transformation through an ecological lens by seeking to understand the contextual factors at multiple levels (*e.g*., consumer, provider, organization) that affect the implementation efforts of treatment innovations into practice through the stages of adoption, implementation and sustainability [15]. IS focuses on preparing and changing the context of practice at multiple levels to accommodate and enhance the fit of the new practice innovation within a particular setting. CA instead takes on a more granular approach focusing on how to make treatments more ecologically valid by systematically considering clients’ and in some cases providers’ , language, cultural values, norms, and meanings, and their context [17]. CA focuses on modifying elements of the EBT without compromising its effectiveness in order to enhance the fit between the treatment and clients’ and providers’ cultural values, preferences and norms.

**Table 1:** Characteristics of implementation science and cultural adaptations

**Characteristics**	**Implementation science**	**Cultural adaptations**
Definition	‘the scientific study of methods to promote the integration of research findings and evidence-based interventions into health care policy and practice’ (PAR-10-038).	“the systematic modification of an evidence-based treatment (EBT) to consider language, culture, and context in such a way that it is compatible with the client’s cultural patterns meanings and values’ [17].
Example of research questions	• How to balance the need to maintain the fidelity of established interventions as they were created, and customize them to local context to increase their relevance, appropriateness, use and uptake?	• What elements of the EBTs need to be adapted to enhance their fit, cultural relevance, and social validity to a specific ethno-cultural group or setting?
	• How to involve and get genuine buy-in and collaboration from multiple stakeholders in the process of implementation?	• How does the culturally adapted EBT retain the active ingredients of the original EBT?
	• How to sustain interventions given constrained financial and human resources and shifting political climates and priorities?	• Will the culturally adapted EBT achieve better client outcomes than the original intervention?
Fidelity perspectives	Balance adaptation and fidelity	Balance adaptation and fidelity
Emphasis of cultural elements	Organizational level and knowledge exchanges between stakeholders	Provider and client levels
Typical unit of analysis	Providers, clinical units, organizations or systems, communities	Patients, families/caregivers, providers
Potential challenges in reducing racial and ethnic disparities in mental health care	• Most implementation trials do not quantify or directly examine their impact in reducing racial and ethnic mental health care disparities	• Culturally adapted EBTs are rarely used in usual care settings
	• Few implementation strategies exist for transporting EBTs in racially and ethnically minority communities	• Culturally adapted EBTs lack explicit attention to implementation context and implementation strategies
	• Most implementation trials do not document the adaptation process when implementing EBT	• Limited evidence that culturally adapted EBTs are more cost- effective than non-adapted EBTs

The fields of IS and CA rarely interact even though both are concerned with making treatments more available and accessible and share the common goal of improving health care in usual care settings. The CA literature rarely acknowledges multi-level implementation factors, particularly at the organization and community levels, but rather has focused on establishing the evidence for the benefits of culturally adapted EBTs. Its emphasis has been on documenting the impact of adapted EBTs on client level outcomes, not on implementation processes or outcomes. The development of the CA literature has been driven in part by the recognition from an influential U.S. Surgeon General’s report more than a decade ago stating that few empirically-supported mental health treatments existed for racial and ethnic minorities since the majority of randomized controlled trials at that time did not include adequate numbers of minorities to establish efficacy and effectiveness in these historically underserved communities [2]. Since then, the CA field in mental health and health has grown and continues to build its evidence base, even though some funding agencies have started to limit funding for studies in this area.

The lack of integration between the IS and CA literature may also be influenced by recent findings from meta-analyses indicating that the efficacy of culturally adapted EBTs is mixed (See Table 2) [18-21]. Culturally adapted mental health treatments produce small to moderate treatment benefits with effect sizes ranging from d = 0.21 to d = 0.46 when compared to an array of controlled/comparison conditions, including un-adapted EBTs, placebo controls, waitlist controls, and/or usual care. However, a closer examination of these meta-analyses indicates that there is great variability in effect size estimates across studies. The heterogeneous nature of study designs, populations, and mental health conditions studied, as well as the type of treatments being compared and adapted, contribute to these divergent findings. Studies included in these meta-analyses are plagued by small sample sizes, and many use outcome measures that lack validity across cultural groups [19].

**Table 2:** Summary of meta-analyses of culturally adapted mental health treatments

**Study**	**Number of studies**	**Type of interventions included**		**Type of study populations**		**Effect sizes**^**a**^				**Significant moderators**
		**Prevention**	**Treatment**	**Children/Youth**	**Adult**	**Culturally adapted vs. heterogeneous controls**^**b**^		**Culturally adapted vs. un-adapted psychotherapy**^**c**^		
						***D***	**95% CI**	***d***	**95% CI**	
Benish *et al*. [18]	59		X	X	X	0.41*	0.38, 0.48	0.32*	0.21, 0.43	• Adaptation to client’s explanatory models
						0.33**	0.13, 0.29	0.21**	0.13, 0.26	
Huey *et al*. [19]	25		X	X		0.44**	0.32, 0.56			• Type of comparison group with largest effect sizes for no treatment control and placebo versus treatment as usual
Griner *et al*. [20]	76	X	X	X	X	0.45^d,^ **	0.36, 0.53			• Age: Older participants had higher effect sizes than younger participants
						0.40^e,^ **	0.30, 0.49			• Hispanic ethnicity: Higher percentage of Hispanic participants had higher effect sizes than studies with lower percentage of Hispanic participants
										• Racially homogenous samples: Studies with racially homogenous samples had higher effect sizes than studies with racially heterogeneous samples
										• Language: Studies that reported language match had higher effect sizes than studies that did not report language match
										• Acculturation: Adaptation seem to benefit most low acculturated Hispanics compared to Hispanics with moderate levels of acculturation
Smith *et al*. [21]	65		X	X	X	0.46**	0.36, 0.56^a^			• Treatment delivered to specific cultural groups were more effective than those delivered to mixed racial/ethnic groups
										• Adapting therapeutic goals to match client’s goals
										• Using metaphors/symbols in therapy to match client’s cultural world views

Note: ^a^All effect sizes reported in the studies reviewed were computed so that positive values indicate greater benefit for culturally adapted treatment over their comparison group; ^b^In these comparisons, culturally adapted treatments are compared to heterogeneous controls conditions that include other un-adapted treatment, usual care, waitlist conditions, and attention control; ^c^In these comparisons, specific culturally adapted psychotherapies are compared to the same un-adapted psychotherapy; ^d^effect sizes for all studies included in this meta-analysis; ^e^effect size for studies that only compared culturally adapted interventions to an ‘alternative intervention.’ *Primary measures; **All measures.

Despite limitations, CA meta-analyses have begun to identify possible moderators that may help clarify what types of treatment adaptations contribute to small to moderate effect sizes for culturally adapted treatments, and which cultural groups may benefit most from adapted EBTs. Both types of moderators can inform the implementation of EBTs in minority communities. For example, the benefits of culturally adapted treatments seem to be driven by treatment modifications related to therapeutic goals, clients’ explanatory models of illness, and the use of metaphor/symbols in treatments that match clients’ cultural world views [18,20]. Cultural adaptations seem to work best for low acculturated Hispanics, non-English speaking clients, older clients, and when treatments are delivered to racially homogenous groups [20]. To advance the implementation of promising EBTs (adapted or un-adapted) in historically underserved minority communities in the U.S., more research is needed to identify key moderators that will help clarify for which populations adapted or un-adapted EBTs work best. Future work should also focus on identifying the types of treatment adaptations that do not dilute the effectiveness of EBTs and produce the most beneficial client and implementation outcomes for underserved populations.

A stronger integration between IS and CA can help address limitations within the two areas. On one hand, IS brings to CA increased attention to multi-level contextual factors that influence how EBTs are used in usual care settings [13] and also expands the focus of CA by considering how the context that surrounds an EBT may need to be adapted to accommodate and enhance the appeal and acceptability of practice innovations [15]. On the other hand, the CA literature can bring to IS field a more nuanced understanding of how clients’ and providers’ cultures influence not only client-level outcomes of EBTs, but also other implementation outcomes, such as the acceptability, accessibility, appropriateness, fidelity and feasibility of EBTs across different stakeholder groups [22]. We propose that the CA literature also contributes with steps and guidelines that can be incorporated into the implementation process to examine how sociocultural factors at multiple levels influence how EBTs are perceived and used by different stakeholders in minority communities [23]. This information can be used to adapt and prepare the context of practice to facilitate the implementation of EBTs. In all, the fields of IS and CA cannot continue to operate in isolation of each other. Their integration can provide an important context for research (See Table 3), producing a more comprehensive approach for considering cultural and contextual forces at multiple levels that influence the implementation of EBTs to address racial and ethnic inequities in mental health care.

**Table 3:** Summary of research implications

**Area of integration**	**Implication for research**
Making culture visible and explicit in the implementation process	• Use CA models to document the process of adaptation and specify what was adapted during the implementation process.
	• Develop user friendly CA guidelines and models that can be used in usual care settings.
	• Integrate CA guidelines and steps into existing implementation strategies.
What to adapt	• Continue to empirically identify the core components of EBTs in order to provide directions for adaptation, if necessary.
	• If adaptations to the EBTs are necessary, CA frameworks can be used to identify what and how elements of the delivery and content of the EBT needs to be adapted to enhance their cultural congruence.
	• Examine how providers’ training should be adapted to increase providers’ adoption of EBT, particularly around issues of cultural competence.
	• Examine how adaptation to the context of practice may facilitate implementation outcomes and help reduce mental health care disparities.
Key players driving cultural adaptations and implementation	• Further specify and document the necessary skills to be an effective facilitator and cultural adaptation specialist.
	• Examine how facilitator and cultural adaptation specialist can collaborate within an implementation team to enhance the implementation of EBTs.
	• Examine how to train a person to incorporate the skills and knowledge of a facilitator and cultural specialist.
Expanding the contextual lens	• Examine how the use of adapted or un-adapted EBTs shown to be effective in racial and ethnic minority communities may enhance providers’ and organizations’ acceptance of EBTs and facilitate their adoption of new practices.
	• Apply methods and steps used in CA models to examine outer contextual factors to gain a deeper understanding of how the local ecology, social norms, and community culture can impact the implementation process.
	• Continue to build the science of CBPR by identifying the core participatory principles and collaborative processes that can facilitate implementation of EBTs in minority communities.
	• Empirically test the effectiveness that participatory approaches compared to other implementation strategies have on implantation outcomes.
	• Examine how different service delivery options can help address workforce shortage issues in historically underserved communities.
	• Cost-effectiveness analyses need to examine whether culturally adapted EBTs result in better outcomes compared to their costs and whether the potential clinical and implementation benefits of culturally adapted EBTs outweigh their costs when compared to un-adapted interventions and usual care across different implementation outcomes.
When to adapt	• Examine how cultural adaptations (if necessary) can be integrated within the implementation process and within existing implementation strategies.

### Making culture visible and explicit in the implementation process

The concept of culture is usually stressed and used in IS at the level of the organization. An organization’s culture ‘essentially what makes that organization unique from all others [24].’ Organizational culture has been defined ‘as the way things are done’ within an organization [25] and it includes values for the services or products provided as well as the interaction between individuals and groups within an organization [24]. Culture at this level has received considerable attention as an influential factor that shapes quality of care and implementation processes [15,25].

Organizational culture has also been addressed in models of knowledge translation, such as the cultural exchange model [26]. Within this paradigm, knowledge translation is conceptualized as the integration and accommodation of the cultural systems (*i.e*., shared understanding, value orientations) that researchers, practitioners, and community members bring to this process [16]. The implementation of EBTs within a community is conceptualized as intercultural encounters where the cultures of stakeholders collide creating a sociocultural event within a social system [27].

Less attention, however, has been given in IS to individual cultural factors at the client and provider levels. An explanation for the limited attention to cultural adaptations at these levels in the implementation field could be due to the general assumption that ‘adaptation happens’ and is considered a natural part of the implementation process [28]. In a recent review of the sustainability of new programs, most studies reviewed indicated that there was some level of adaptation during the implementation and sustainability of the intervention. However, virtually no study provided descriptions of what was adapted or the explicit process used to adapt the intervention [29]. We advocate, therefore, that teams involved in adapting interventions document their adaptations during the implementation of EBTs and make explicit the process and methods used to make these adaptations. Such documentation of the adaptation process may have several benefits. It may help generate more generalizable knowledge about the type of adaptations that produce better implementation and treatment outcomes in a new context. It can also help clarify processes, steps and methods used to adapt EBTs to enhance their fit with a new population and/or context. In all, the careful and explicit documentation of the adaptation process can help produce a knowledge base to guide future implementation efforts.

Cultural issues at the client and provider levels should be visible in the implementation of EBTs, as ignoring these cultural elements may lessen the impact, relevance, acceptability and sustainability of promising EBTs when transported to usual care settings, particularly those serving racial and ethnic minority communities [9]. The use of existing CA models can provide guidelines and methods for making culture more visible in the implementation process [23]. Such models include recommendations that treatment adaptations: (a) be informed by the expertise of stakeholders (*e.g*., researchers, clients, providers, administrators) and include collaborations between treatment developers, treatment users, and community members; (b) use formative research methods (*e.g*., focus groups, surveys, in-depth interviews) to understand population needs, risk and resilience factors, and the context of practice; (c) make efforts to modify and document the process of engagement and retention of participants, including attention to acceptability of the treatment; (d) consider treatment provider and/or therapist characteristics as a central component of the intervention by focusing on the ecological validity of the intervention; (e) address the fidelity-adaptation tension via iterative pilot testing to refine adaptations; and (f) conduct formal evaluations of the adapted intervention to test its effectiveness [30-32].

CA models have been used to adapt and evaluate a number of EBTs, such as cognitive behavioral therapy [33,34], interpersonal psychotherapy [35], and parent management training [36]. CA models provide general steps and guidelines for identifying and specifying how culture influences different EBTs without compromising their effectiveness [17]. CA models, however, have emerged within the context of efficacy and effectiveness trials; thus, their utility in usual care settings is unknown and untested. Further research is needed on how to make CA models user friendly within the realities of usual care practice settings with limited resources, time and expertise, and how to integrate them into existing implementation strategies.

### What to adapt: balancing adaptation and fidelity

Both the literature of IS and CA address the degree to which adaptation may be required to enhance the transportability of an EBT without diluting its effectiveness [9,37]. Given the potential transformative nature of the implementation process, some level of adaptation to the local context is likely to occur as most EBTs are usually not ‘street ready’ for real-world settings [38]. When implementing EBTs, therefore, researchers and providers face the tension between implementing a standardized treatment as designed versus adapting it to the target context or population. The balance between these two ends of the debate will likely affect the implementation, dissemination and sustainability of the intervention [39]. For example, adaptations can be made to the delivery or to the content of the intervention, and it can be driven by the organizational context and by providers’ and clients’ behaviors [40]. There are several approaches that may be considered in order to achieve a balance between adaptation and fidelity by identifying what to adapt in the delivery, content of the intervention, or the context that surrounds the intervention.

One approach is to consider the core functional elements and forms of treatments. From this perspective, the core functional elements of a treatment (i.e., its active ingredients)-are retained in the implementation process, while the forms by which this treatment is delivered (*e.g*., group vs. individual sessions) are customized to local contingencies [41]. This approach may be most useful when treatment developers have empirically identified the intervention’s core elements in order to provide guidance to local implementers on how to tailor the different forms of treatment without diluting essential treatment mechanisms [37]. For many EBTs, however, the core elements, including delivery methods, have not been empirically identified or supported and developers may not be amenable to change their protocols without this level of evidence.

This approach also lacks specification for identifying what to adapt, particularly the specific intervention elements necessary to make EBTs more culturally responsive to ethno-cultural groups. Resnicow and colleagues [42] address this limitation by specifying two key levels for informing cultural adaptations*:* surface adaptations and deep adaptations. Surface adaptations involve customizing the intervention materials and messages to the ‘observable’ social and behavioral characteristics of a target population [42]. These adaptations focus on making peripheral aspects of the intervention fit with the culture, experience, and behavioral patterns of a particular group in order to enhance the intervention’s appeal and face validity [43]. Examples of surface adaptations involve customizing audiovisual or print materials by changing pictures of people, places and language to be more aligned with patient or client culture. For example, surface adaptations may be necessary when delivering an intervention originally developed in one language or culture to a new language or cultural group. For some interventions, surface adaptations may be sufficient, but for others, deep level adaptations may need to be considered [42].

Deep adaptations involve a reflection on how ‘cultural, social, psychological, environmental, and historical factors influence health behaviors differently across racial/ethnic populations’ [42]. Deep adaptation may posit differences in the mechanisms of change of the intervention and incorporates culture-specific conceptualizations of the problem (*e.g*., explanatory models of illness), social norms (*e.g*., gender roles), and cultural beliefs into the intervention in order to enhance cultural sensitivity and create the conditions for the desired behavioral change [44]. In summary, surface adaptations address basic understanding and perception of materials, whereas deep adaptations convey the cultural salience and impact of the content and skills the intervention aims to provide.

Another useful framework that can help identify what to adapt is the Ecological Validity Model [45] based on the ecological systems theory [46]. This model proposes eight domains (language, persons, metaphors, content, concepts, goals, methods, and context) that one should consider in order to enhance the cultural congruence between participants and the intervention. Based on this framework, intervention adaptations should ensure that the language, metaphors, and methods used to communicate and deliver the treatment message and technology are understandable and culturally appropriate [36]. Attention to the persons involved in the treatment should focus on the cultural values, norms and expectations that shape interpersonal dynamics and preferences between clients and therapists. Adaptation should also attend to the content and goals of treatments that must reflect and respect participants’ cultural knowledge, values, norms, and traditions, and, if possible, support adaptive values and behaviors from the client’s culture of origin.

Adaptations can also be made to intervention content and to approaches used to train providers. While providers’ attitudes toward EBTs generally tend to be positive, some providers may have doubts about the effectiveness of the EBTs [47] or, if they have positive attitudes towards the science behind the EBT, they tend to be concerned about the impact that the use of standardized manuals have on their performance with their clients or their discretion in applying treatments [48-51]. Providers may perceive that some EBTs and their manuals or guidelines have a tendency to ‘overlook client’s family needs, culture, and strengths’ [52,53], affecting both provider and client engagement in the intervention.

Another challenge providers face when working with diverse populations is how to engage and retain clients through appropriate application of the intervention [54,55]. The type, content and approaches used to train providers are important variables that can affect the uptake of EBTs [56]. Modifying providers’ trainings to highlight the intervention components that can be modified to incorporate providers’ clinical expertise, and to train providers in methods and community outreach approaches that can facilitate recruitment and engagement of culturally diverse clients, may allow for a flexible approach that could increase providers’ uptake of EBT without diluting its reach and effectiveness [54,57]. Ongoing coaching after training may also improve providers’ acceptance and adherence to the intervention while also preventing intervention drift [58].

Lastly, the context that surrounds the intervention may provide important targets for adaptation. From an IS perspective, Aarons and colleagues [59] suggest that modifications may need to be made to the organizational context (*e.g*., resource, provider training, organizational culture) to facilitate the fit of the EBT to the practice setting. Contextual factors at the provider and organizational levels influence racial and ethnic disparities in mental health care and play a pivotal role in the implementation of EBTs in these communities [1,2,60]. Examples of organizational level adaptations that can help reduce mental health care disparities include: increasing the availability of bilingual providers and/or trained interpreters, expanding hours of operation, changing client flow protocols to reduce waitlist, and expanding the delivery of services to community settings frequented by minority clients. These types of adaptations focus on modifying organizational elements to improve the accessibility, responsiveness and feasibility of mental health care, all of which are critical for reducing mental health care disparities.

### Key players driving cultural adaptions and implementation

During the implementation process, IS studies have shown that the use of a facilitator, a person who applies tailored strategies to enable change within a complex system in order to promote the implementation of the intervention, is a promising implementation strategy [61,62]. Although there is a lack of clarity about the roles of the facilitator [63], Kauth and colleagues indicated that the facilitator is the person ‘engaged in a formal implementation process, focused on knowledge application in specific settings’ [62]. Other key characteristics of the facilitator are that he/she should be credible, persuasive, engaging, provide education, set goals for change, assess progress and barriers to implementing the intervention, tailor actions to local context, develop supportive relationships with stakeholders, assist in problem solving, and provide individualized feedback to providers [62]. Facilitators can come from within or outside of the organization. Studies suggest that the combination of external and internal facilitators seems to be most effective [63,64].

It is not clear, however, whether or not, or how the facilitator works with the treatment developers or research team to increase the fit between the intervention and the target population. Such is the role of the “cultural adaptation specialist” (CAS) used in some cultural adaptation models. The CAS has knowledge about the target population and the population for which the intervention was originally developed in order to help researchers and practitioners incorporate core elements of the target culture in the adapted intervention [31]. In general, the CAS should: (a) be familiar with the target community, its values and practices beyond language competence (*e.g*., knowing how to speak Spanish is not enough to be considered culturally competent when working with Latino communities); (b) be self-aware of his or her values; (c) actively participate in the community; (d) use conceptual frameworks to inform intervention adaptations in order to make them culturally responsive; (e) be knowledgeable about the intervention; and (f) have problem solving skills to be able to bridge information between the target population and the research team.

The skills required to be a facilitator and a CAS are different. The CAS is more focused on the target population rather than on the provider. There is also no explicit mention in adaptation models about whether the CAS should know how to negotiate the larger context, such as system or organizational factors. The role of the facilitator, on the other hand, emerged as a method for encouraging intervention uptake by providers in clinical practice [63] and has not necessarily focused on the fit of the intervention with the target client or patient population.

Considering that the abilities of the CAS and facilitator are different in scope, the integration between these two roles requires further study. One alternative could be to train a given person to have the skills and knowledge of both a facilitator and CAS. For example, facilitators could attend cultural competency trainings to assist on the adaptation of the intervention to the target population. Several models of cultural competency training have been developed and are being used as strategies to improve providers’ skills in addressing the unique needs of ethno-cultural groups [65]. Alternatively, a CAS could be trained as a facilitator by gaining knowledge about organizational level issues known to impact the implementation process.

Care should be taken, however, regarding the selection of the key players who will have the role of CAS, facilitator, or both. In particular, much discussion in the field of CA cautions against having a single person, particularly a mental health professional or expert, holding the role of the cultural gatekeeper of the community. Community members should be included and engaged in the process of selecting and adapting an EBT, as they bring an insider’s perspective on the needs, preferences, barriers, and the potential response the community may have towards the EBT [66,67]. Moreover, as far as training of the CAS, discussion in the field still exists as to whether this person should be from academia (which some authors advocate against), from the community, or represent and straddle both worlds [10].

A more realistic alternative, therefore, could be to have multiple key players collaborating in the adaptation and implementation efforts. In fact, research in implementation science is showing that transdisciplinary collaboration among scientists and stakeholders from different disciplines and communities are needed for the effective implementation and sustainment of interventions [68]. Both CAS and facilitator, therefore, could collaborate during the implementation process using collaborative models such as team science [69] or community-based participatory research (CBPR) approaches [66,70]. In summary, research is needed to delineate the tasks and skills of the CAS and the facilitator, and how to best incorporate them into the implementation process.

### Expanding the contextual lens

Implementation does not happen in a vacuum; it is embedded within a broader ecological context that goes beyond client and provider factors and impacts all aspects of the implementation process [71,72]. Cultural adaptation models usually fall short in informing the implementation process because they tend to ignore important contextual factors that impact how EBTs are used and sustained in organizations and community settings [22]. Expanding the contextual lens, as commonly used in IS studies, and integrating them with principles and lessons from CA models can help advance how promising EBTs are transported, used and sustained to address racial and ethnic disparities in mental health care. Context is commonly defined as ‘the set of circumstances or unique factors that surround a particular implementation effort’ [13], including the physical environment, policies, infrastructure, and the social and political forces that shape this change process [73]. A common model for understanding and conceptualizing context in IS is to consider the inner and outer context that surrounds the EBT [13,15].

The inner context encompasses factors within the organization where the implementation of an EBT takes place. Common inner contextual factors include: the structures, social context, and leadership of organizations. Organizational structures are the attributes (*e.g*., size, mission, services, workflow, location, etc.) that characterize an organization and have been found to either support or hinder the implementation process [60]. The organization’s social context includes the climate and culture of the organization and the work attitudes of the people within the organization [24,74]. Each of these elements could impact client level outcomes and quality of care [24]. Findings from organizational level interventions [75,76] that target the social context indicate that the implementation of EBTs could benefit by planned, systematic and customized approaches that accompany the EBT to remove inner contextual barriers and create the organizational conditions that support the use of the EBTs in community settings.

The outer context that surrounds the organization also plays a critical role in the implementation of EBTs. Common outer contextual factors in IS include: community cultural norms and attitudes toward particular health and mental health conditions and their treatments, community resources and capital, and policies and political forces. Mendel and colleagues defined community norms and attitudes as: ‘a range of attitudes and knowledge about particular health conditions, expectations and priorities toward types of treatments or client populations, and collectively held beliefs and values that may affect the receptivity of individual and organizational stakeholders to adopt or adhere to a new care practice or intervention’ [60]. Given the combination of stigma towards mental illness in the U.S. [77] and generally low levels of health literacy in racial and ethnic minority communities [78], community norms and attitudes likely play an important role in the implementation of EBTs. Attitudes ‘set the context in which individuals in the community respond to the onset of mental health problems, clinicians respond to individuals who come for treatment, and public policy is crafted’ [79]. Ignoring these community level issues may be detrimental to the introduction and acceptance of an EBT, as it may be in direct conflict with local cultural understandings and practices for addressing health and mental health conditions and can result in resistance and failure [27]. In the same way that the culture of clients and providers needs to be made visible in the implementation of EBTs, the community’s cultural norms and attitudes in the outer context should be taken into consideration. Norms and attitudes can influence how mental health problems are perceived and discussed among community stakeholders, how their support is sought, and what types of EBTs may be considered acceptable and worthy of implementation. A mismatch between the EBT, the process of implementation, and stakeholder cultural norms may create resistance and even deep suspicions about why EBTs are being introduced and implemented [27]. The application of methods used in CA models (*e.g*., focus groups, involvement of community members) to assess the outer context could be used to gain a deeper understanding and appreciation of the local ecology, social norms, and community culture in order to facilitate the implementation of EBTs in a specific community. Such methods could be used to help customize the EBT to these community characteristics and also harness community assets, capabilities and strengths for EBT implementation efforts [27,60].

CBPR approaches provide another viable avenue to address these community-level issues that arise as EBTs are introduced and implemented in new communities. CBPR can enhance the implementation process by contextualizing EBTs to the cultural and social realities of communities, integrating ethno-cultural values, perspectives and preferences into the content and form of EBTs to enhance their relevance, acceptability and feasibility, and by strengthening the capacity of community stakeholders to support implementation efforts and community-based research to reduce mental health care disparities [66,70,80].

The integration of CBPR and IS is a fertile area for future research, particularly in identifying the core participatory principles and collaborative processes that facilitate the implementation of EBTs. More research is also needed to empirically test the effectiveness of participatory approaches compared to other implementation strategies on implementation outcomes. A recent example of this head-to-head comparison in the U.S. is the integration of CBPR principles and approaches within a large randomized implementation trial, the Community Partners in Care [81]. This trial is comparing the impact that two implementation approaches, a CBPR community engagement strategy and a professional technical assistance strategy, have in the implementation and outcomes of evidence-based depression care in communities of color in Los Angeles, California. More studies of this nature are needed to advance the science of CBPR and IS in reducing mental health care disparities.

Another critical set of outer context factors includes the resources and capital that can support or thwart implementation efforts. Resources include financial, human, social, cultural, and political capital that exist in organizations and in the community [60]. For example, many communities (*e.g*., rural, reservations, frontier towns) lack the appropriate workforce to deliver mental health services, and consequently tend to modify the delivery method (*e.g*., increase the group size of interventions or shorten sessions), which could affect the effectiveness of the intervention [82]. A critical consideration for implementation efforts in these communities could be to expand the repertoire of service delivery options, such as the use of telemedicine, paraprofessionals, and community health workers, to address workforce issues.

The scarcity of human and financial resources for delivering mental health services, particularly in urban and rural minority communities in the U.S., demands that a business case in which the economic benefits exceed the cost of implementation be established in order for stakeholders to support the investment and introduction of a new practice innovation [1,2]. This business case should consider other costs and benefit issues, such as the intervention’s acceptance and compatibility with the client population, which could enhance treatment engagement and reduce treatment drop outs. This is difficult given that many EBTs, particularly culturally adapted EBTs, typically lack cost and cost-effectiveness data. More studies are needed to document the direct and indirect costs and benefits for the introduction, use and sustainment of an EBT within minority communities. Cost-effectiveness analyses should also examine whether culturally adapted EBTs result in better outcomes compared to the costs of un-adapted interventions and usual care.

Lastly, policies and political forces, such as regulatory practices, legal liabilities, funding mechanisms, and reimbursement regulations have a profound impact on the implementation and sustainment of EBTs [15,83]. Policies can shape the type, forms and frequency of practices that can and cannot be implemented and can create incentives and disincentives for organizations and communities to engage and invest in the implementation of EBTs [60]. They can be used as leverage points throughout the entire ecology of implementation, imparting their influence at the levels of the organization, regulatory agencies, and the political and social milieu [83,84]. It is not sufficient for EBTs to be culturally and linguistically appropriate for racial and ethnic minorities; the context that surrounds an EBT must be carefully considered in order to enhance its uptake, and ultimately its impact in reducing mental health care disparities [22].

### When to adapt in the implementation process

Client, provider, and larger contextual factors shape the implementation of EBTs, and accommodation to the local ecology is an important step in the implementation process. A critical question, therefore, is: When should adaptations to the EBT, if necessary, occur during the implementation process?

Several cultural adaptation models provide guidance. For instance, Kumper and colleagues stipulate that adaptations should follow only when there is empirical evidence from pilot implementation studies and from clients and providers’ feedback that the original intervention does not fit the new population’s needs [10]. Moreover, Lau’s selective and directed cultural adaptation framework provides a set of guidelines to decide when to customize treatments to particular client populations [85]. Based on this data-driven model, adaptations are warranted when there is quantitative and/or qualitative evidence that: the new population has distinctive and unique sociocultural context of risk and resilience that will require consideration or addition of new treatment elements to address these contextual issues; and/or when the social validity of an intervention is compromised, thus limiting treatment engagement and the receipt of an adequate exposure to the treatment to achieve its intended effect. Barrera and colleagues expand upon Lau’s model and propose further guidelines for determining when adaptations may be justified early on in the implementation process. They stipulate that adaptations are needed when qualitative and/or quantitative evidence indicates that two or more populations differ on: treatment engagement dimensions (*e.g*., awareness of treatment, treatment completion); the ability of the treatment to change mediating variables (action theory); and the relationships between mediators and treatment outcomes (conceptual theory) [86].

In both models, adaptations occur early on in the implementation process, particularly when deciding which EBTs to implement (Exploration phase) and preparing for the adoption of the new treatment (Preparation phase) to the new settings and populations [82]. These two models are useful for determining when EBTs need to be adapted to client characteristics, but provide little guidance for deciding when adaptations to other important elements (*e.g*., service context, organizational characteristics) of implementation should be considered and how to incorporate these adaptations throughout the implementation process.

Adaptations to EBTs when necessary should not be a separate or an additional step in the implementation process. Rather, adaptations need to be considered as the process of adding, omitting, or modifying elements of the intervention and, therefore, an implementation outcome, along with other outcomes such as fidelity and dosage [32]. In fact, several recent IS models currently being tested provide operational guidelines and steps for incorporating adaptation decisions within the implementation EBTs in new settings and diverse populations [39,87]. For example, the Dynamic Adaptation Process (DAP) model specifies a process for identifying and incorporating adaptations at multiple levels and throughout the phase of implementation to facilitate the adoption and use of EBTs in community settings [82]. Within DAP, adaptation decisions are made by an implementation resource team (IRT) composed of multiple stakeholders (*e.g*., clinicians, researchers, administrators, clients, intervention developers). The IRT uses information from a careful assessment of system, organizational, provider and client level characteristics to negotiate system, organization and intervention adaptations while maintaining core ingredients of the EBT. Adaptations within this model may include re-ordering, forestalling or delaying, de-emphasizing, emphasizing or augmenting EBT components. The DAP model considers adaptations beyond the EBT, such as modifications to the service context or the organization itself to facilitate implementation. The DAP also includes a feedback process that includes fidelity checks and quality assurance procedures during the implementation phase to guide, monitor and address system, organization and EBT adaptations while trying to achieve a high level of fidelity to the core elements of the EBT. In summary, the models described above support the need for adaptation processes to be planned and started in the early stages of implementation, but also recognize that adaptations are likely to be needed throughout the implementation process and can include adaptation to the EBT as well as system, organization, and practice context.

## Discussion

In this paper, we have argued for a stronger integration between the IS and CA fields that can support implementation of the best available EBTs for historically underserved communities in the U.S. to reduce disparities in mental health care. An important contribution of CA models to IS is to make cultural issues, particularly at the client and provider levels, more visible in the implementation process. If clients and providers can understand and identify with the EBT they should be more likely to see its benefits and understand its activities, and accept and benefit from the intervention [88]. If culture is not addressed at these levels - EBT implementation is less likely to be successful, no matter how well the EBT fits with the organizational culture and policies.

Furthermore, CA and IS can provide balance in the need to retain the active ingredients of a treatment to maintain its effectiveness while customizing treatments to improve their fit in specific contexts. Such a balancing act can be complicated by limited resources, local expertise, and capacity that organizations and researchers face when deciding what to adapt in the implementation process. No clear answers currently exist regarding how to achieve optimal balance, but the blending of IS and CA perspectives can help specify what needs to be adapted in order to enhance EBT implementation. Explicit documentation through the use of quantitative and/or qualitative methods can provide insights into dynamic implementation processes and clarify not only how adaptations occur but at what level, for what purposes, and how they may impact implementation and client outcomes.

Who facilitates and drives the implementation process is an important consideration for the implementation of EBTs in underserved minority communities. Collaborative approaches (*e.g*., team science, CBPR) that include the facilitator and cultural adaptation specialist (CAS) may be the most productive, as each brings different skills and knowledge to the implementation process. Examples of questions that require future work in order to integrate these two roles within the implementation of EBTs include: What are the unique tasks and skills of a facilitator and a CAS? What are the best strategies to train one person as a facilitator and CAS? What are the most effective collaborative approaches to integrate these two roles within an implementation project?

A contribution that IS can offer CA is the attention to inner and outer contextual factors that impact how EBTs are ultimately used and sustained in real-world settings. This contextual lens is also critical for the development and use of implementation strategies [89,90], which are systematic processes and actions (*e.g*., academic detailing, quality management, learning collaborative) used to adopt and integrate EBT into community settings [91]. The assessment and identification of contextual factors through qualitative and quantitative methods can provide the basis for developing customized implementation strategies to accompany EBTs. These contextual assessments can be incorporated into the adaptation process and/or included as elements of existing cultural adaptation frameworks as a way to bridge the fields of IS and CA. The development and testing of implementation strategies linked to specific EBTs to reduce racial and ethnic mental health care disparities is a fertile field for future research.

Finally, when to adapt is still an open question that requires further empirical work. Due to the dynamic nature of the implementation process, it seems that the adaptation process should also be dynamic and done at different points in time during the implementation process. To help identify when should adaptation occur, feedback mechanisms should be in place throughout the process to make the necessary adjustments to the adaptation and implementation of the EBT. Careful documentation and tracking of the adaptation processes, therefore, can help clarify when adaptation happens, how adaptation decisions are made, who facilitates the adaptation, and when they are most valuable during the implementation process. These are still open questions for the field, and more studies are needed to test which adaptation models produce the best implementation results in historically underserved minority communities. In all, developing a two-way street between IS and CA can provide a better avenue for moving the best available treatments into practice and help in the reduction of racial and ethnic disparities in mental health care.

## Abbreviations

CA: Cultural adaptations; CAS: Cultural adaptation specialist; CBPR: Community-based participatory research; DAP: Dynamic adaptation process; EBT: Evidence-based treatment; IS: Implementation Science.

## Competing interests

LJC and AAB declare that they have no competing interests.

## Authors’ contributions

Both authors contributed to the development of this paper. LJC had the original ideas for this paper. LJC and AAB collaborated in expanding the ideas for this paper, reviewing the literature and writing drafts. Both authors approved the final version of the paper.
